# Comment on: Comparison of four surgical approaches for rectal prolapse: multicentre randomized clinical trial

**DOI:** 10.1093/bjsopen/zrac042

**Published:** 2022-03-21

**Authors:** Steven R. Brown, Asha Senapati

**Affiliations:** 1 Dept of Surgery, University of Sheffield, Sheffield, UK; 2 St Marks Hospital, London, UK

*Dear Editor*

The Swedish rectal prolapse trial has been a long time in reporting^1^. It finished recruiting over 12 years ago and took 9 years to recruit 134 participants (just over one patient/centre/year). Despite protracted recruitment time, it was still under-recruited and did not achieve an adequately powered study based on the outcome of recurrence.

Although this may be considered a significant failing, one should not necessarily be too critical. A concurrent trial with a comparable design had the same issues. The PROSPER trial also ran for 7 years despite 34 centres. Interestingly, the average recruitment/centre/year was also around one.

It is clear from these two well-supported trials that any randomized comparative trial on rectal prolapse is destined to fail for reasons the investigators of both trials discuss. Indeed, this protracted recruitment resulted in both trials becoming redundant before they had finished as laparoscopic ventral mesh rectopexy was not included as an intervention. An alternative study design is required if the holy grail of recurrence is to be decisively defined. And yet funders of such alternative study designs are resistant, certainly in the UK. Perhaps the Danish can find a way with their recently funded Nordic Rectal Prolapse Trial based on an observational cohort with an enhanced design.

The conclusions of both trials are the same. Namely quality of life improves whatever the intervention. Given the similarity of study design, it is valid to combine data for recurrence. Although still underpowered, such an analysis suggests recurrence at 3 years is high with all interventions and again shows no difference between comparisons. Recurrence is 27 per cent after abdominal approach *versus* 30 per cent after perineal surgery (odds ratio (OR) −0.08 (95 per cent confidence interval (CI) −0.32, 0.15)), 38 per cent after Delormes *versus* 30 per cent after Altemeier (OR 0.69 (95 per cent CI 0.40, 1.17)), and 11 per cent after resection rectopexy *versus* 24 per cent after suture rectopexy (OR 0.41 (95 per cent CI 0.14, 1.17)).

## References

[1] J. Smedberg , W. Graf, K. Pekkari, F. Hjern. Comparison of four surgical approaches for rectal prolapse: multicentre randomized clinical trial. BJS Open 2022;6:zrab140 10.1093/bjsopen/zrab140 PMC8769527 35045155

